# Structured Waters Mediate Small Molecule Binding to G-Quadruplex Nucleic Acids

**DOI:** 10.3390/ph15010007

**Published:** 2021-12-22

**Authors:** Stephen Neidle

**Affiliations:** The School of Pharmacy, University College London, 29-39 Brunswick Square, London WC1N 1AX, UK; s.neidle@ucl.ac.uk

**Keywords:** G-quadruplex, ligands, naphthalene diimides, acridines, crystal structures, water clusters, hydrogen bonding

## Abstract

The role of G-quadruplexes in human cancers is increasingly well-defined. Accordingly, G-quadruplexes can be suitable drug targets and many small molecules have been identified to date as G-quadruplex binders, some using computer-based design methods and co-crystal structures. The role of bound water molecules in the crystal structures of G-quadruplex-small molecule complexes has been analyzed in this study, focusing on the water arrangements in several G-quadruplex ligand complexes. One is the complex between the tetrasubstituted naphthalene diimide compound MM41 and a human intramolecular telomeric DNA G-quadruplex, and the others are in substituted acridine bimolecular G-quadruplex complexes. Bridging water molecules form most of the hydrogen-bond contacts between ligands and DNA in the parallel G-quadruplex structures examined here. Clusters of structured water molecules play essential roles in mediating between ligand side chain groups/chromophore core and G-quadruplex. These clusters tend to be conserved between complex and native G-quadruplex structures, suggesting that they more generally serve as platforms for ligand binding, and should be taken into account in docking and in silico studies.

## 1. Introduction

G-quadruplexes (GQs) are higher-order four-stranded structures that can form in DNA and RNA sequences by the folding of repetitive short guanine (G)-tracts [1,2,3,4]. These are typically interspersed with short runs of general sequence. The G-tracts self-associate by Hoogsteen hydrogen bonding to form guanine (G)-quartets, several of which stack on one another to form a G-quadruplex (GQ). The quartets are held together by the phosphodiester backbones, and by the general sequences, which typically form extra-helical loops. GQ folding and hence the nature of the loop can occur in several ways, such that the four strands can be all-parallel, all-anti-parallel, or various combinations [2,5]. GQ prevalence in the human genome is non-random [6,7]. In eukaryotic cells, they occur in telomeres [8,9] and are over-represented in genomic promoter [10,11,12,13,14] and untranslated sequences [15,16,17], especially in genes and pathways involved in cancer initiation and progression [14,18,19,20,21,22]. GQ-forming sequences have been found in the genomes of many organisms, ranging from viruses [23,24,25,26,27] to bacteria [28,29,30,31] and malaria [32,33,34]. GQs have been visualized in fixed [35,36,37,38] and in live cells [39,40,41,42], where their existence may be more than transient, with several roles in gene function [3,4,12]; for example, GQ folding is associated with sites of active transcription and precedes transcription itself [13,22]. 

The presence of GQ sequences in the promoters of oncogenes, such as h*TERT*, *MYC*, *KRAS*, *BCL2*, and c-*KIT* as well as their potential for destabilizing telomere maintenance in cancer cells (and their involvement in replication and genomic instability [12,43,44,45]), has focused attention on GQs as promoter or telomeric therapeutic targets in viral and bacterial diseases as well as human cancers. The strategy of stabilizing them with appropriate small-molecule compounds has resulted in many chemically diverse chemotypes being investigated, notably against oncogene GQ promoter targets [12,46,47]. Formation of high-affinity GQ-ligand complexes within promoters has been demonstrated to reduce or even abolish transcription of a target gene or genes, see for example refs. [48,49,50,51,52,53,54,55,56,57,58], which can result in cell growth inhibition and in vivo anti-cancer activity in animal models of human cancers [59].

**Figure 1:**
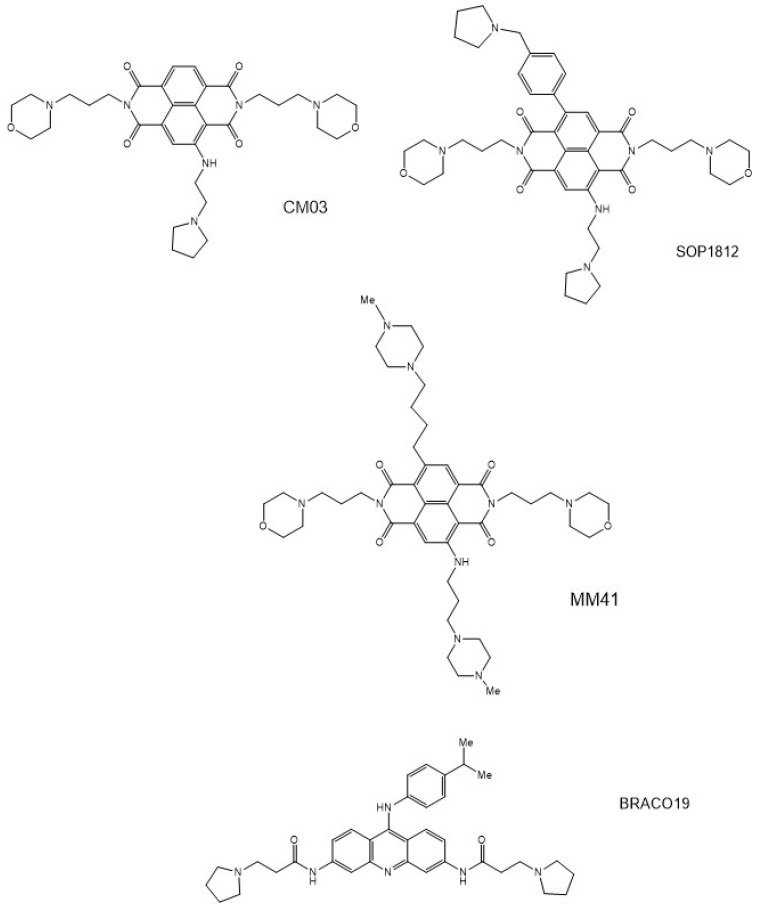
Structures of the tri-substituted naphthalene diimide derivatives (CM03), two tetra-substituted derivatives (MM41 and SOP1812), and the trisubstituted acridine compound BRACO19. CM03: 2,7-bis(3-morpholinopropyl)-4-((2-(pyrrolidin-1-yl)ethyl)amino) benzo[lmn][3,8] phenanthroline-1,3,6,8(2H,7H)-tetraone; SOP1812: 2,7-bis(3-morpholinopropyl)- 4-((2-(pyrrolidin-1-yl)ethyl)amino)-9-(4-(pyrrolidin-1-ylmethyl)phenyl) benzo[lmn][3,8] phenanthroline-1,3,6,8-(2H,7H)-tetraone); MM41: (4,9-bis((3-(4-methylpiperazin-1-yl)propyl)amino)-2,7-bis(3-morpholinopropyl) benzo[*lmn*][3,8] phenanthroline-1,3,6,8(2H,7H)-tetraone); BRACO19: 3,6-bis(3-pyrrolidin-1-ylpropionamido)-9-(4-dimethylaminophenylamino) acridine.

Most of these GQ-binding compounds share common structural features of planar heteroaromatic groups and side chains carrying cationic groups, albeit in a wide variety of chemotypes [60,61,62,63,64,65,66]. Structure-activity studies have optimized activity for numerous series of these compounds, for for example several libraries of acridine [67,68,69,70,71,72,73,74,75,76] and naphthalene diimide [18,19,77,78,79,80,81,82,83,84,85,86,87,88,89,90,91,92,93,94] derivatives. The lead naphthalene diimide derivatives CM03 and SOP1812 (Figure 1) show evidence of target engagement and in vivo anti-tumor activity [18,19]. Crystallographic and NMR studies have provided detailed information on GQ-ligand interactions in these and other GQ-ligand complexes [50,51,71,74,75,76,80,81,95,96,97,98,99,100], some of which have been used in several hit-to-lead optimization projects [18,19,50,81,90,97,99]. Quantitative and semi-quantitative computer modeling methods including docking procedures [101,102,103,104,105,106,107,108,109,110,111] have also been extensively employed to screen virtual libraries, with the aims of aiding optimization and identifying plausible new chemotypes and lead compounds for future drug development and eventual clinical trial.

Several high-resolution crystallographic studies of native GQs [112,113,114,115] and GQ-ligand complexes [71,74,75,80,81,98,100,116,117] have reported that water molecules are intimately associated with GQ sites and with bound ligand. It is well-established for DNA-, RNA- [118], and protein-ligand complexes [119,120,121,122,123] that water molecules can play critical target-ligand mediation roles. The present work analyzes water arrangements in several GQ-ligand complexes (PDB id 3CE5, 3NZ7, and 3UYH) [74,81,100] that are of sufficient resolution to give confidence in the significance of the arrangements, to better understand the role and the consequences of discrete water molecules associated with ligand binding. The naphthalene diimide complex [81] was also chosen since it involves the tetra-substituted compound MM41 (Figure 1). This is the direct precursor of two other more recently designed naphthalene diimide compounds, CM03 and SOP1812 [18,19] (Figure 1), which are currently in pre-clinical development stages. Hydration features of this complex and other complexes examined here, it is suggested, have wider implications for GQ-based drug design and hit/lead selection.

## 2. Results

Only the crystal structures containing acridine, berberine, and naphthalene diimide (ND) derivatives fulfilled the acceptance criteria summarized in the preceding section. Two co-crystal structures are available (Table 1) for the tetrasubstituted naphthalene diimide compound MM41 (Figure 1) complexed to intramolecular human telomeric GQs, for which it has high binding affinity. Structure PDB id 3UYH is at the higher resolution of the two and consequently a greater number of ligand-associated water molecules were observed in electron density maps and included in the final refined crystal structure [81]. Hence it was chosen for further detailed analysis (Table 1 and Table 2). Structure 3CDM [124] has 2 G-quadruplexes, 158 water molecules, and 4 substituted naphthalene diimides in the asymmetric unit, i.e., 79 waters per G-quadruplex. However, few water molecules in this structure are resolved in the vicinity of the two stacked naphthalene diimide ligands compared to structure 3UYH and so this structure was not chosen for detailed analysis. In addition, the nature of naphthalene diimide substituents in 3CDM is not directly relevant to MM41 and hence not to CM03 or SOP1812, so this structure was not considered any further in the present analysis. 

### 2.1. MM41 Side Chain Contacts and Water Environment

**Figure 2:**
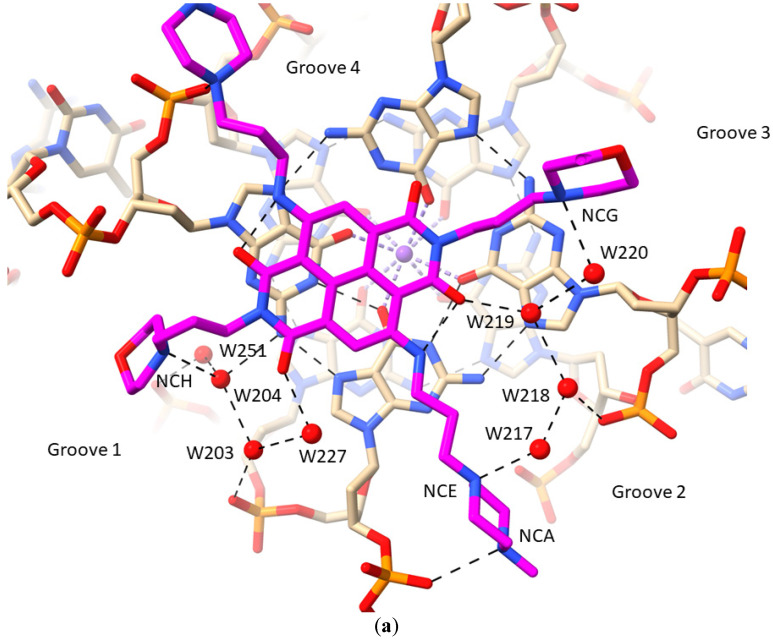

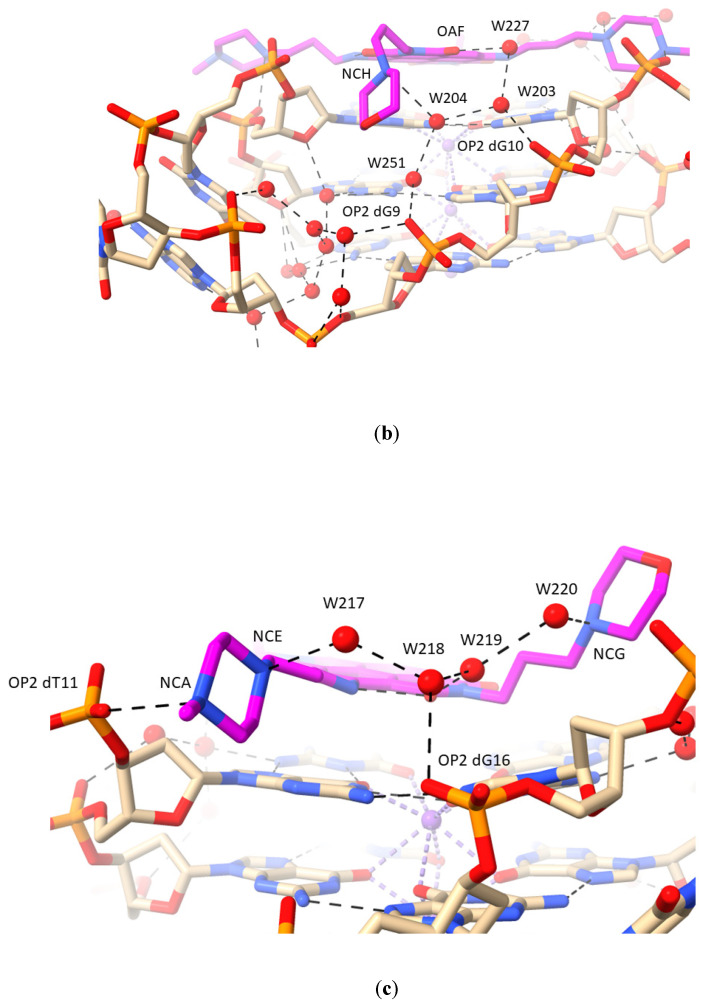
Views of the crystal structure of MM41 (with its carbon atoms colored magenta) bound to a human intramolecular telomeric G-quadruplex, PDB id 3UYH. (**a**) The view is projected onto the G-quartet plane and shows the extent of overlap with the naphthalene diimide core chromophore. The water molecules that are in direct or indirect contact with the MM41 molecule, are shown as red spheres, with hydrogen bonds indicated by dashed lines. This and the subsequent figures were drawn using the ChimeraX package (https://www.cgl.ucsf.edu/chimerax/, last accessed on 16 December 2021) [125]. (**b**) A view of the 3UYH complex looking into groove 1. The four water molecules are shown that are hydrogen bonded to the morpholino group in this groove and the OAF carbonyl oxygen atom of the naphthalene diimide core. A nearby cluster of water molecules is also shown, embedded deep in the groove and adjacent to a TTA loop. (**c**) A view of the 3UYH complex midway between grooves 2 and 3, highlighting the group of four water molecules hydrogen bonding to an N-methyl-piperazine and a morpholino group in these grooves.

MM41 has two side chains terminating in N-methyl-piperazine groups and two with terminal morpholino groups. Each of these groups can be assumed to be protonated at physiological pH, with N-methyl-piperazine having a pK of 8.5 compared to the slightly less basic morpholino group, with a pK of 9.2 [81]. Figure 2a shows a view of structure 3UYH projected onto the planes of the G-quartets and the naphthalene diimide core, highlighting the grooves of the GQ. Each MM41 side chain is positioned in or close to the mouth of a GQ groove, although only three of the four end groups are actually situated within a groove. The fourth, having a terminal morpholino ring, is oriented away from the quartet plane and a detailed examination of the crystal structure has indicated that rotation of the side chain to place the morpholino group into groove 4 is sterically hindered by the small surface area of the naphthalene diimide core compared to that of the quartet [81].

All four basic end groups of MM41 have their protonated nitrogen atoms in hydrogen bond/electrostatic contact with atoms in the GQ grooves (Table 2). However, only two of these contacts, each involving the terminal nitrogen atom of an N-methyl-piperazine group, has a direct nitrogen-phosphate group hydrogen bond interaction (N…OP distances of 2.9 and 3.1 Ǟ). The three other end groups all have water contacts with ring nitrogen atoms (Figure 2b,c), which in the case of the morpholino groups, are presumed to be protonated. Groove 1 (Figure 2b) has the morpholino group positioned at the mouth of the groove. A small linear cluster of four water molecules extends from the morpholino basic nitrogen atom, with one water contacting two further waters, which contact with two neighboring phosphate oxygen atoms. One of these waters OP2 dG10 also contacts a fourth water molecule, which in turn contacts the adjacent oxygen substituent on the naphthalene diimide chromophore. The other water, contacting a phosphate oxygen atom (OP2 G9), also contacts and is thus the link to a water molecule in a second water network that fills the rest of this groove. The N-methyl-piperazine group in groove 2 (Figure 2c), which is situated at the mouth of the groove, has its terminal nitrogen atom (NCA) in close contact with a phosphate oxygen atom, OP2 dT11, suggesting that this nitrogen atom carries a proton. The inner piperazine ring nitrogen atom contacts another linear group of four water molecules which extends into groove 3 and terminates with a hydrogen bond contact with the second morpholino group. The second water in this array has a contact with a phosphate oxygen atom (OP2dG16), and the third is in hydrogen bond contact with carbonyl oxygen atom OAG of the ND core. The second N-methyl-piperazine ring, also situated at the mouth of groove 4, has a direct contact involving the outer ring nitrogen and a phosphate oxygen atom (Figure 2a), but does not have any associated water molecules.

**Figure 3:**
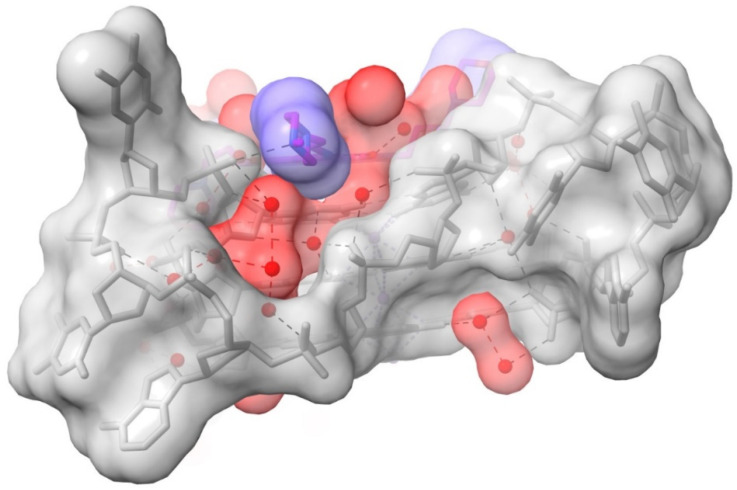
Surface representation of a view into groove 2 in the 3UYH complex. The N-methyl-piperazine substituent of MM41 is oriented end-on and is colored blue. Note that the groove space is filled out by water molecules, colored red. The semi-transparent surface of the G-quadruplex is colored grey.

The above section describes water molecules involved in ligand contacts; other waters fill out the remaining space in the grooves (the non-completeness in some grooves is most likely due to limitations of the crystal structure at 1.95 Ǟ with only a fraction of the potential total number of water molecules located in electron density). Groove 2 is one of the more completely resolved grooves in terms of hydration (Figure 3), with waters embedded deep into the groove. This complex array of 12 water molecules, of which the majority are first-shell, hydrogen bond with phosphate oxygens, O4′ and O5′ atoms, and guanine base edges (which form the floor of the groove). The net effect is to maintain the relative positions of TTA loop and the groove.

### 2.2. MM41 and Water Mobility

It is notable that there is remarkably little overlap between the ND four-ring core and the individual guanines in the top quartet, as seen in Figure 2a. The surface area of the ND core of MM41 is too small to allow simultaneous overlap with more than one guanine of the top G-quartet. Computational experiments were undertaken using two different energy minimization protocols (in the ARGUSLAB and AVOGADRO packages) to assess the effect of removing all the water molecules from the structure. Minimization in both cases resulted in movement of the ND core by ca 1.5 Å to produce improved overlap with a guanine base of the G-quartet. The cationic groups in the side chains tended to move closer to the phosphate oxygen atoms. Attempts to dock the MM41 molecule onto the water-free GQ using AutoDock Vina 1.1.2 as installed within the database G4LDB 2.2, resulted in a series of almost equi-energetic poses in which three out of four side chains were positioned away from the grooves. This was not pursued further.

The side chain heterocyclic groups in MM41 have greater mobility than the ND core, as revealed by their individual atomic temperature factors (see the PDB entry for 3UYH and Table 2). The five nitrogen atoms in these terminal rings, which are hydrogen-bonded to waters or phosphate groups, have a mean B value of 51 Ǟ^2^, corresponding to a <U> of 0.8 Ǟ. The water molecules in the mini cluster around and contacting the morpholino ring in groove 1 have lower B values, with most in the range 27–32 Ǟ^2^, corresponding to a <U> of 0.6 Ǟ. The cluster of waters in groove 2/3 have slightly greater mobility.

**Figure 4:**
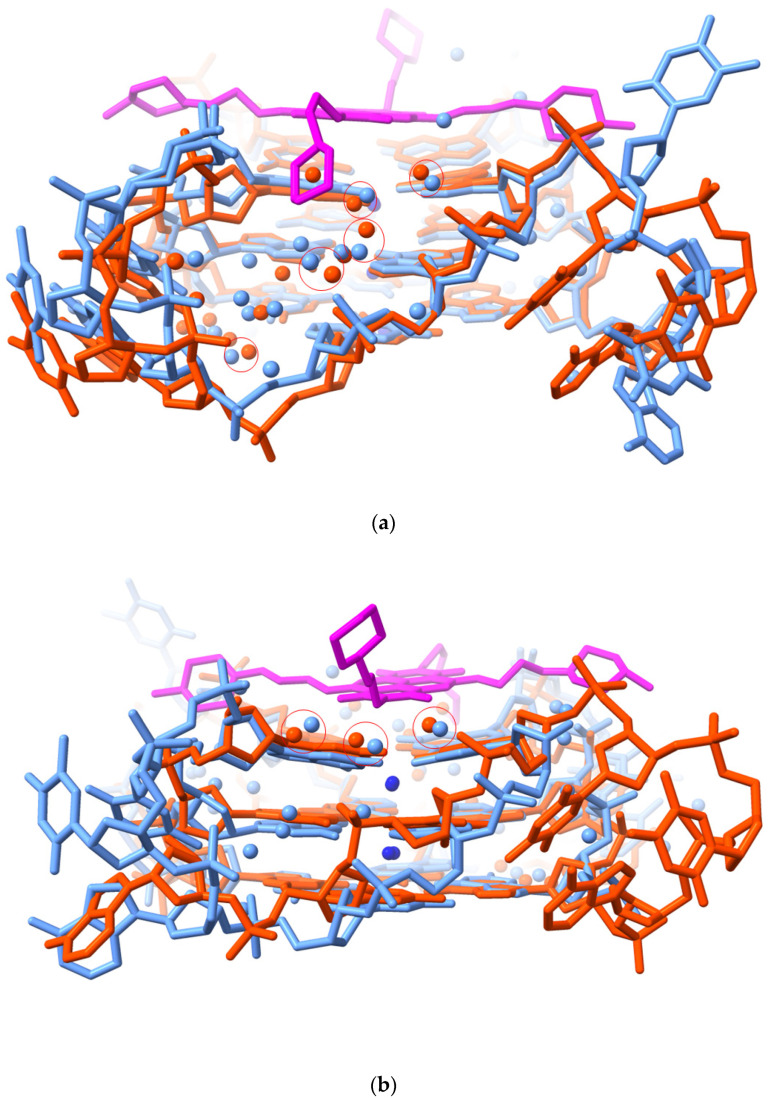
Superposition of the G-quartets of native and MM41-complexed G-quadruplex crystal structures, 1KF1 and 3UYH, respectively, viewed into groove 1. The native structure is colored light red, the ligand-bound is cyan, and the MM41 molecule is shown magenta. Only the water molecules in the groove are shown, colored as in their G-quadruplex structures. Those water molecules in the two structures that are <1.0 Å to each other are enclosed in red circles. (**a**) Viewed into groove 1. (**b**) Viewed into groove 3.

The extent to which water molecules located in the 3UYH crystal structure correspond to those found in the native structure was examined by superimposing on 3UYH the native crystal structure 1KF1 [126], overlaying the G-quartets (Figure 4a,b). Overlap of the quartets was good, as expected. However, the loops in the MM41-bound structure adopt distinct conformations compared to those in the native structure. Systematics of loop conformations in GQ crystal structures have been previously reported [95] and will not be further discussed here. Detailed comparison of water positions revealed that the cluster of four waters at the mouth of groove 1 that mediate between ono the morpholino end groups of MM41 and G-quadruplex is also present in the native structure (Figure 4a), with distances between each pair of waters (i.e., a 3UYH water ….1KF1 water) 0.6–1.0 Ǟ. Since these waters are mobile with a <U> of 0.6 Ǟ, they can be considered to likely occupy the same space. Three conserved water molecules are also present at the mouth of groove 3 (Figure 4b), close to the second morpholino end group.

### 2.3. Water Mediation in Acridine-G-Quadruplex Structures

**Figure 5:**
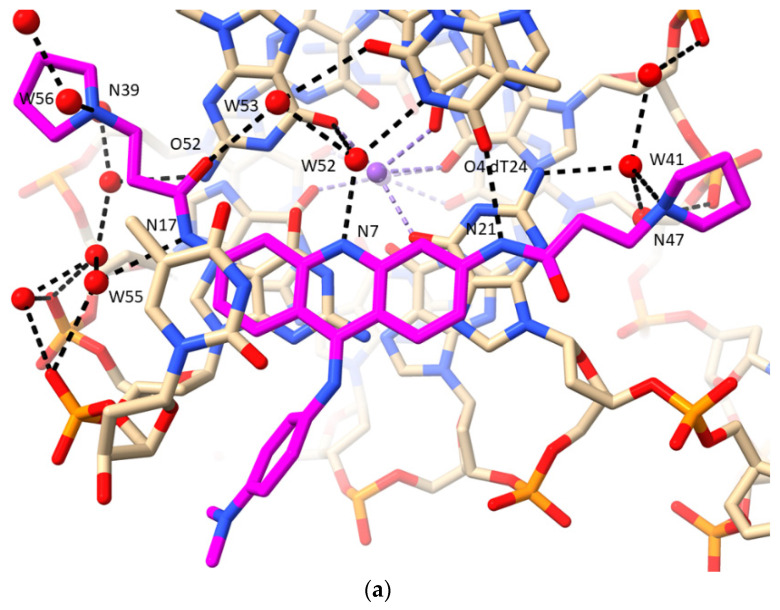

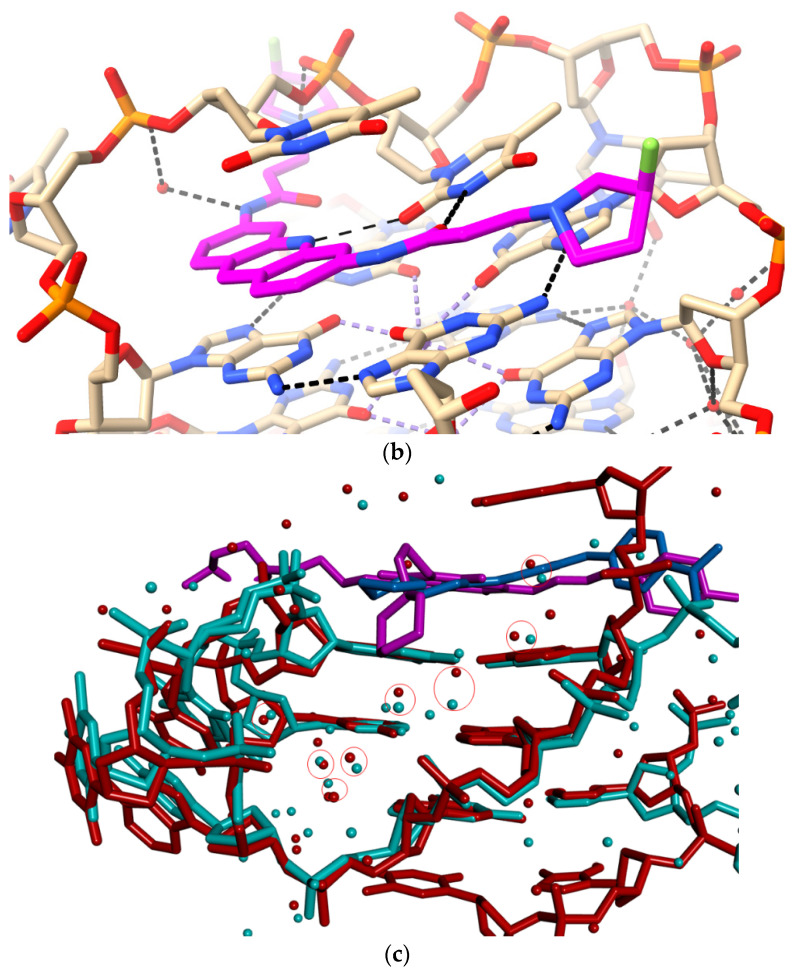
(**a**) View of the BRACO19 complex with a human telomeric bimolecular G-quadruplex, as observed in the crystal structure PDB id 3CE5 [74], projected onto the acridine plane. The carbon atoms of the ligand are colored magenta and water molecules are colored as red spheres. Hydrogen bonds are shown as dotted lines. Some water molecules not directly involved in ligand interactions have been omitted from this view to enhance clarity. (**b**) View of the complex involving a disubstituted acridine with a fluorine atom attached to each terminal side chain pyrrolidino ring, bound to an *Oxytricha nova* bimolecular G-quadruplex [100]. Color coding is as in other figures, with the fluorine atoms colored yellow. (**c**) Overlay of structures 3UYH (G-quadruplex in cyan and MM41 in magenta) and 3CE5 (G-quadruplex in red and BRACO19 in dark blue), superimposed on the G-quartets, viewed into groove 1 of the 3UYH structure. Water molecules in the groove are colored as in their G-quadruplex structures. Those water molecules in the two structures that are <1.0 Å distance to each other are enclosed in red circles.

The crystal structure (PDB id 3CE5) of the complex between the experimental drug BRACO19 and a bimolecular human GQ [74], shows that, in common with the MM41 complex, the GQ has adopted a parallel topology. In both instances, the ligand is stacked onto one end of the GQ, onto a terminal G-quartet. The BRACO19 molecule has three cationic charges at physiological pH, one in each side chain pyrrolidino ring and one on the central ring nitrogen atom in the acridine ring. None of these are directly hydrogen-bonded to anionic phosphate groups. Instead (Table 2 and Figure 5a), they are hydrogen bonded to water molecules. The waters hydrogen bonded to the pyrrolidino cationic nitrogen atoms do eventually link indirectly to phosphate groups, via further water molecules. Water molecule W52 hydrogen bonded to the acridine central ring nitrogen, appears to play a crucial role, hydrogen bonding both to an O6 of a guanine from the adjacent stacked G-quartet, and to N3 of a thymine in-plane with the acridine. W52 also hydrogen bonds to W53, which in turn hydrogen bonds to the carbonyl oxygen atom of one of the amide side-chains on BRACO19. The other amide group is trans to this and its amide nitrogen atom hydrogen bonds to O4 of this thymine—this is the sole direct drug—GQ hydrogen bond, with the remaining five being water-mediated. An overlay of the MM41 and BRACO19 complexes (Figure 5c) shows that several of the key water molecules are conserved between the two structures, with distances between pairs of waters < 1 Å, using the argument outlined in the previous section.

Removal of the water molecules from this structure followed by energy minimization, resulted in movement of the acridine by ca 2.5 Å, enabling the acridine ring nitrogen atom to directly contact the thymine ring substituents. Such an arrangement has been observed in the series of co-crystal structures [75] involving disubstituted acridines with the bimolecular anti-parallel GQ from *Oxytricha nova* (Table 2). These structures, exemplified by the two high-resolution structures [100] with fluorine substituents in the pyrrolidino side chains, have direct O_2_ thymine and N3 hydrogen-bond contacts with the acridine ring and an amide carbonyl oxygen atom (Figure 5b). A water molecule is involved in mediating between a side chain amide and a phosphate oxygen atom. However, the *Oxytricha nova* complexes all have a distinct anti-parallel GQ topology, with the acridine constrained within a tetranucleotide diagonal loop, with little room for any associated water molecules.

## 3. Discussion

The number of water molecules located in nucleic acid and protein crystal structures is invariably less than the total present in the crystal lattice. Thus, crystal structures 3UYH and 3CE5, whose reported water molecules are analyzed here, both have an estimated 56% solvent, which corresponds to >400 water molecules. A small fraction of this, 51 and 54 water molecules, respectively, were observed in electron density maps [74,81]. These are almost all first or second shell immobilized waters. 

Three principal findings emerge from the current analysis:

1. The morpholino end groups of MM41, which are assumed to be basic in the buffering conditions of the crystallization experiment and in biological solution, do not directly contact the GQ. Hydrogen bonding/electrostatic interactions with negative backbone phosphate groups were anticipated but were not observed. Instead, the basic ring nitrogen in each morpholino group hydrogen bonds to one of a group of four water molecules positioned in the mouth of the relevant grooves (1 and 3). The waters are in hydrogen bond contact with backbone phosphates. Similarly, the basic pyrrolidino side chain terminal groups of BRACO19 do not directly contact phosphate groups in its GQ complex, with water mediation being observed in the crystal environment.
2. A nitrogen atom on both N-methyl-piperazine groups of MM41, by contrast, directly hydrogen bonds to a backbone phosphate oxygen atom, implying greater basicity than morpholino for this end group.
3. The water clusters associated with the two morpholino groups of MM41 are highly conserved between the native and the MM41-bound GQ structures. There is also conservation of a number of the ligand-associated waters between the MM41 and BRACO19 structures, and by implication, between the native and BRACO19 structures.

We suggest that the conserved water clusters have relevance to the observed structure-activity relationships for MM41 derivatives [81]. Thus, replacing the morpholino groups with isosteric groups such as hexose or ether groups, which lack the morpholino hydrogen bonding ability, results in an almost complete loss of GQ affinity and reduced biological activity compared to MM41. It is also notable that none of the four side chains are deeply embedded in the GQ grooves, and one might have therefore expected reduced GQ affinity compared to analogues with longer side chains. This is not the case since, as observed here, the short side chains are effectively captured by hydrogen bonded to the conserved water clusters, which would have to be displaced by longer side chains. This would be at a significant entropic cost. The strategy of replacing two of the four strongly basic end groups characteristic of earlier ND compounds (see, for example refs. [55,57,79]), by less strongly basic morpholino groups [18,19,81], has culminated in the design and evaluation of MM41, and subsequently compounds CM03 and SOP1812 (Figure 1), which are currently being assessed as pre-clinical candidates. The rationale for lowering the highly cationic nature of these ND compounds is that this could improve cellular uptake and tumor distribution while retaining GQ affinity. The present analysis has shown that this substitution has preserved the water structure around the perimeter of the grooves in which the morpholino groups bind, with no diminution of GQ affinity [90].

The mediated waters in the BRACO19 structure also have relevance to the observed structure-activity relationships for BRACO-GQ interactions [73,74], and for BRACO19 biology. Although BRACO19 may not be further developed as an anti-cancer drug [127], its activity against a variety of anti-viral GQ targets [128,129,130] suggests the likelihood of further anti-viral analogue programs, for which the 3CE5 structure and its structured water features are of direct relevance.

The present analysis, although limited to two crystal structures, demonstrates that water molecules can play an active role in GQ-ligand recognition. This indicates that in silico and docking studies of ligand-GQ binding need to take account of reliably located explicit water molecules. It is concluded that their omission will lead to misleading conclusions on low-energy ligand binding states and interactions. Many such studies still tend to ignore the role of water and really require input from high-resolution crystal structures or reliable and well-validated water modelling/simulations. This has been recognized in several studies for example, refs. [118,131]. Prediction of water positions and mobilities in ligand complexes can be made using molecular dynamics [131], although this has only rarely been been used to date for GQ systems [132]. The prediction of water positions having low mobility in nucleic acids by use of a specially generated water force field together with statistical scoring has led to the development of an automated method, termed “SPLASH’EM” (Solvation Potential Laid around Statistical Hydration on Entire Macromolecules) [118], which has given results for duplex DNA and some RNA structures in good agreement with experiment. It will be interesting to see this type of approach used for those GQ ligand complexes for which there is high resolution structural data, as well as therapeutically important GQ drug targets such as that from the KRAS promoter sequence [114]. Conserved groove water molecules have been identified in the grooves of this crystal structure [114], as well as in other high-resolution GQ native structures [115]. By analogy with structures 3UYH and 3CE5, these conserved and structured waters should be retained in docking studies. The present analysis indicates that such water platforms for ligand binding can form an essential part of the total low-energy GQ interaction complex.

## 4. Materials and Methods

Crystal structures were downloaded from the Protein Data Bank and visualized by the ChimeraX (https://www.cgl.ucsf.edu/chimerax/, last accessed on 16 December 2021) [125] and BIOVIA Discovery Studio (https://www.3ds.com/products-services/biovia/, last accessed on 16 December 2021) programs.

Criteria for further consideration of individual structures were:

1. Resolution ≤ 2.5 Å,
2. Having at least one water molecule contacting a ligand,
3. Hydrogen bonds were accepted in a structure if

donor-acceptor distances ≤3.25 Å
donor-hydrogen…acceptor angles were ≤30° from ideality, and

4. Relevance to current drug discovery.

We excluded the daunomycin complexes with d(G_4_) and d(TG_4_T) (PDB ids 3TVB and 1O0K) from consideration, even though they are of high resolution and have large numbers of localized water molecules [116,117]. Their relationship to human GQ ligand complexes is unclear, because of their characteristics of multiple bound and stacked daunomycin molecules.

Root-mean-square displacement (RMSD) values, <U> (=<U^2^>)^1/2^, in Å, were calculated from the deposited experimental crystallographic isotropic temperature factors (B factors in Å^2^), using the relationship

<U> = (B/8π^2^)^1/2^

B factors, obtained from the refinement of a crystal structure, indicate the relative isotropic thermal motions of individual atoms in a crystal structure.

Molecular mechanics and docking studies were performed on structure PDB id 3UYH using the ARGUSLAB (http://www.arguslab.com/arguslab.com/Publications.html. Last accessed on 23 August 2021) and AVOGADRO (https://avogadro.cc/, last accessed on 23 August 2021) packages. The UFF Universal Force Field [133] was used in these calculations. Docking studies were also undertaken within the G-quadruplex ligand database G4LDB 2.2 (https://www.g4ldb.com. Last accessed on 16 December 2021) [134], which utilizes the docking modules in AutoDock Vina 1.1.2 [135].

## 5. Conclusions

This study has analysed data from earlier crystal structure analyses and has shown that 1st and 2nd shell water molecules play an important role in the binding of two experimental small-molecule drugs, MM41 and BRACO19 to human telomeric G-quadruplexes. These waters mediate between cationic side-chain functional groups and phosphate backbones. They also directly bridge the chromophore core of the drugs and other G-quadruplex groups. Altogether, waters serve to maintain the drug molecules in their low-energy binding positions and their removal would result in incorrect drug positions. This has implications for drug design and virtual library screening and docking.

## Figures and Tables

**Table 1:** G-quadruplex-small molecule crystal structures, taken from the Protein Data Bank, for which at the minimum a first shell of water molecules around the DNA and ligand have been reported. The number of water molecules associated with each complete G-quadruplex is quoted, as taken from the PDB entry. The structures discussed here (PDB ids 3CE5, 3NZ7 and 3UYH) are highlighted in bold.

PDB Id	G-Quadruplex Type	Compound	Resoln (Å)	No. of Waters/AU	Ref.
**3CE5**	**12-mer bimolecular human telomeric**	**3,6,9- trisubstituted acridine BRACO19**	**2.5**	**54**	**74**
**3NZ7**	**12-mer bimolecular *Oxytricha nova* telomeric**	**3,6- disubstituted acridine, F substituents**	**1.10**	**187**	**100**
3NYP	12-mer bimolecular *Oxytricha nova* telomeric	3,6- disubstituted acridine, F substituents	1.18	176	100
3EM2	12-mer bimolecular *Oxytricha nova* telomeric	3,6- disubstituted acridine	2.3	64	75
3EQW	12-mer bimolecular *Oxytricha nova* telomeric	3,6- disubstituted acridine	2.2	66	75
3EUI	12-mer bimolecular *Oxytricha nova* telomeric	3,6- disubstituted acridine	2.2	159	75
3ERU	12-mer bimolecular *Oxytricha nova* telomeric	3,6- disubstituted acridine	2.0	71	75
3ES0	12-mer bimolecular *Oxytricha nova* telomeric	3,6- disubstituted acridine	2.2	56	75
3ET8	12-mer bimolecular *Oxytricha nova* telomeric	3,6- disubstituted acridine	2.45	51	75
3EUM	12-mer bimolecular *Oxytricha nova* telomeric	3,6- disubstituted acridine	1.78	52	75
1L1H	12-mer bimolecular *Oxytricha nova* telomeric	3,6- disubstituted acridine	1.75	146	75
**3UYH**	**22-mer human telomeric**	**Tetrasubstituted naphthalene diimide MM41**	**1.95**	**51**	**81**
3T5E	22-mer human telomeric	Tetrasubstituted naphthalene diimide BMSG-SH-4	2.10	38	80
3CCO	11-mer biomolecular human telomeric	Tetrasubstituted naphthalene diimide	2.20	28	124
3CDM	22-mer human telomeric	Tetrasubstituted naphthalene diimide	2.10	158	124
4DA3	21-mer human telomeric	Tetrasubstituted naphthalene diimide MM41	2.40	25	81
6S15	12-mer bimolecular human telomeric	Pyridine derivative of berberine	1.70	23	98

**Table 2:** Hydrogen bond interactions. (**a**) In structure 3UYH, involving the tetrasubstituted naphthalene diimide MM41, a human intramolecular telomeric G-quadruplex, and water molecules. Hydrogen-bond distances are shown (d_1-2_ in Å), together with the reported crystallographic B factor values (in Å^2^) for MM41-bound waters and associated MM41 and DNA atoms. MM41 atoms are highlighted in bold red type. Waters in direct contact with MM41 atoms are highlighted in bold blue type. Numbering is as in the PDB entry. (**b**) In structure PDB id 3CE5 [74], involving the trisubstituted compound BRACO19, a human intermolecular bimolecular telomeric G-quadruplex, and water molecules. Parameter definitions and color coding are as in Table 2a.

(a)					
	Atom_1_	Atom_2_	d_1-2_	B Factor Atom_1_	B Factor Atom_2_
Groove 1					
	**NCH**	**W204**	3.2	**64**	**32**
	**W204**	W203	3.0	**32**	32
	W203	OP2 dG10	2.9	32	32
	W203	**W227**	3.4	32	**45**
	**W227**	**OAF**	2.5	**45**	**29**
	W251	OP2 dG9	2.9	27	30
	**W204**	N2 dG4	2.9	**32**	20
	**W204**	W251	2.8	**32**	27
Groove 2					
	**NCA**	OP2 dT11	3.1	**46**	35
	**NCE**	**W217**	2.7	**40**	**46**
	**W217**	W218	2.6	**46**	48
	W218	**W219**	3.0	48	**43**
	**W219**	**ODX**	2.7	**43**	**31**
	W218	OP2 dG16	3.4	48	45
Groove 3					
	**NCG**	**W220**	2.9	**48**	**50**
	**W219**	**W220**	2.9	**43**	**50**
Groove 4					
	**NCF**	OP2 dG4	2.9	**58**	39
**(b)**					
	**Atom_1_**	**Atom_2_**	**d_1-2_**	**B Factor Atom_1_**	**B Factor Atom_2_**
Groove 1					
	**N7**	**W52**	2.7	**11**	**21**
	**W52**	N3 dT24	2.9	**21**	12
	**W52**	O6 dG5	3.0	**21**	18
	**W52**	**W53**	3.1	**21**	**33**
	**W53**	O2 dT24	3.3	**33**	18
	**W53**	**O52**	3.1	**33**	**14**
Groove 2					
	**W56**	**N39**	3.4	**23**	**27**
	**N17**	**W55**	3.0	**14**	**38**
	**W55**	O2 dT12	3.3	**38**	17
Groove 4					
	**N21**	O4 dT24	3.0	13	17
	**N47**	**W41**	3.0	**21**	**28**
	**W41**	W44	2.9	**28**	29
	W44	OP2 dG23	2.5	29	21
	**W41**	N2 dG17	2.9	**28**	18

## Data Availability

Data is contained within the article.
